# Polyarylene Ether Nitrile and Barium Titanate Nanocomposite Plasticized by Carboxylated Zinc Phthalocyanine Buffer

**DOI:** 10.3390/polym11030418

**Published:** 2019-03-04

**Authors:** Shuning Liu, Chenchen Liu, Changyu Liu, Ling Tu, Yong You, Renbo Wei, Xiaobo Liu

**Affiliations:** Research Branch of Advanced Functional Materials, School of Materials and Energy, University of Electronic Science and Technology of China, Chengdu 611731, China; liushuningg@126.com (S.L.); liuchenchen@std.uestc.edu.cn (C.L.), 13086660861@163.com (C.L.); tutu576695@163.com (L.T.); yourkeaib@163.com (Y.Y.)

**Keywords:** barium titanate, polyarylene ether nitrile, zinc phthalocyanine, dielectrics, rheology

## Abstract

Barium titanate (BT) and polyarylene ether nitrile (PEN) nanocomposites with enhanced dielectric properties were obtained by using carboxylatedzinc phthalocyanine (ZnPc-COOH) buffer as the plasticizer. Carboxylated zinc phthalocyanine, prepared through hydrolyzing ZnPc in NaOH solution, reacted with the hydroxyl groups on the peripheral of hydrogen peroxide treated BT (BT-OH) yielding core-shell structured BT@ZnPc. Thermogravimetric analysis (TGA), transmission electron microscopy (TEM), TEM energy dispersive spectrometer mapping, scanning electron microscopy (SEM), X-ray diffraction (XRD), X-ray photoelectron spectroscopy (XPS), and Fourier transform infrared (FTIR) demonstrated successful preparation of BT@ZnPc. The fabricated BT@ZnPc was incorporated into the PEN matrix through the solution casting method. Rheological measurements demonstrated that the ZnPc-COOH buffer can improve the compatibility between BT and PEN effectively. With the existence of the ZnPc-COOH buffer, the prepared BT@ZnPc/PEN nanocomposites exhibit a high dielectric constant of 5.94 and low dielectric loss (0.016 at 1000 Hz). BT@ZnPc/PEN dielectric composite films can be easily prepared, presenting great application prospects in the field of organic film capacitors.

## 1. Introduction

To fulfil demands from up-to-date electronic industry, high dielectric permittivity material has shown its essentiality in the fields of capacitors, electromagnetic interference shielding materials, and energy storage fields. [1,2,3] Comparing with the traditional high dielectric ceramic materials including barium titanate (BaTiO_3_), piezoelectric ceramic transducer lead zirconatetitanate etc., [4,5], the polymer dielectric materials are widely used in energy storage appliances owing to their light weight, flexibility, and easy processing, which show the suitability for the lightweight devices and miniaturization [6,7,8]. The polymeric dielectrics have been widely used in the capacitors and other electronic devices. Up to now, polyvinylidene fluoride (PVDF), polyethylene (PE), and epoxy resin are often used as polymer dielectric [9,10,11]. However, these polymers often demonstrate poor heat resistance, which lead the adding of cooling device and the high cost of the appliance [12] when using them as the dielectrics. Polyarylene ether nitrile (PEN), belonging to the aromatic ring polymer, is a new type of polymer exhibiting temperature, radiation and chemical resistance, and mechanical properties as well as insulation [13,14,15]. PEN also shows low processing temperature, great temperature resistance, and short molding period compared with polyetheretherketone (PEEK) [16,17]. These advantages facilitate the processing of PEN into all kinds of forms to fulfill the dielectric applications. 

The defect of the polymer dielectric materials is their relatively low dielectric permittivity. In comparing with the permittivity of the ceramic dielectric which can be hundreds or even thousands, the permittivity of polymer dielectrics is usually below 15 [13]. For example, the permittivity of PE is only ~2.0, and it is ~3.3 for epoxy resin. Although the existing of plenty of cyano groups in the system, the permittivity of PEN is only about 4.2 [14]. To solve this problem, high permittivity fillers and/or conductive fillers are usually introduced into the polymer substrate offering polymeric composite dielectric materials [18,19]. The polymeric composite dielectric materials can combine excellent properties and are lightweight from the polymer substrate, with high permittivity from the fillers. Unfortunately, most of the fillers are inorganic materials, which show poor compatibility with the polymer matrix. This will further result in the phase separation between the polymer matrix and the fillers which elevates the dielectric loss simultaneously [19,20]. Surface modification of the fillers with organic groups is an effective method in fabricating polymeric composite dielectric materials with high permittivity and low dielectric loss. 

In this paper, the BT as the filler and PEN as the polymeric substrate were employed to fabricate polymeric composite dielectric materials. To improve the compatibility between the polymer matrix and the filler, BT was wrapped with carboxylatedzinc phthalocyanine (ZnPc-COOH), which is compatible with the PEN matrix offering BT@ZnPc. The SEM, TEM, TEM EDS Mapping, XRD, FTIR, and XPS were used in characterizing composition of BT@ZnPc. Thereafter, BT@ZnPc were incorporated into the PEN substrate to prepare the BT@ZnPc/PEN film. The compatibility between the PEN matrix and the BT@ZnPc was investigated through rheology measurements and the dielectric property of the obtained polymeric composite dielectric materials was further studied.

## 2. Materials and Methods

### 2.1. Materials

**Scheme 1:**
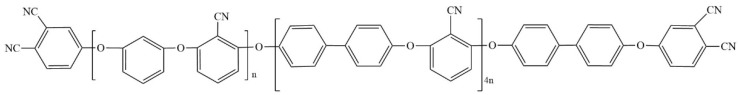
Structure of polyarylene ether nitrile (PEN).

BT was bought from Aladdin, Shanghai, China. Hydrogen peroxide (H_2_O_2_), tetrahydrofuran (THF), and *N*-methyl-2-pyrrolidone (NMP) were supplied by Tianjin Bodi Chemicals, Tianjin, China. PEN (Scheme 1) was polymerized through nucleophilic aromatic substitution polymerization according to the procedure reported in the literature [21,22]. ZnPc was synthesized by a zinc chloride catalyzed self-cyclization reaction of diphenyl bisphthalonitrile in an NMP solution [23,24].

### 2.2. Core-Shell Structured BT@ZnPc

Hydroxylated barium titanate (BT-OH): BT-OH was prepared according to the previous literature [25]. BaTiO_3_ (8.0 g) as well as H_2_O_2_ (200 mL) were added into a 500 mL flask, then the mixture was stirred and heated under reflux for 6 h, and finally the BT-OH was obtained through filtering. It was further purified through washing by water several times and drying in a vacuum oven at 80 °C for 24 h. 

Carboxylated zinc phthalocyanine (ZnPc-COOH): ZnPc (2.3 g) and 240 mL of 1.0 mol/L NaOH solution were added into a 500 mL flask. The mixture was heated under reflux for 8 h. Then, 500 mL of 1.0 mol/L HCl solution was added into the ZnPc solution in batches. After stirring for 24 h at room temperature (RT), ZnPc-COOH was obtained by filtering. ZnPc-COOH was purified by washing with water to neutral and dried in a vacuum oven at 80 °C for 24 h. 

BT@ZnPc: 2.0 g of BT-OH was ultrasonically stirred in 80 mL NMP for 1 h, then an appropriate amount of ZnPC-COOH was put in. This system was agitated under RT with ultrasonication for 3 h. After that, this mixture was sealed by an autoclave, and then this system was kept at 200 °C for 4 h. The obtained outcome was filtered, washed by water, and dried in a vacuum oven at 80 °C for 24 h. The core-shell structured BT@ZnPc with different organic layer thicknesses was prepared and named as BT@ZnPc-1, BT@ZnPc-2, and BT@ZnPc-3, respectively, by adding 0.4, 0.6, and 0.8 g ZnPC-COOH during the preparation process.

### 2.3. BT@ZnPc/PEN Nanocomposites

BT@ZnPc/PEN nanocomposite films were obtained by solution casting method. BT@ZnPc/PEN nanocomposites with 0, 5, 10, 20, and 30 wt% BT@ZnPc, respectively, were homogeneously dispersed in NMP through ultrasonication at 80 °C for 1 h. Afterwards, this mixture cast on a horizontal glass was dehydrated under a preset program. Nanocomposite film was obtained after cooling down to room temperature. BT/PEN film was similarly fabricated through the same procedures for comparison.

### 2.5. Characterization

Scanning electron microscopy (SEM) was applied in investigate microstructures of BT, BT@ZnPc, and BT@ZnPc/PEN using a JEOL JSM-5900LV (Tokyo, Japan). Transmission electron microscopy (TEM) micrographs were gained by a ZEISS Libra 200 FE (Oberkochen, Germany) at 200 kV. XRD analysis were achieved via the Bruker D8 ADVANCE A25X (Karlsruhe, Germany) under Cu Kα radiation. FTIR measurements and XPS were applied to determine the structure of zinc phthalocyanine by using a Shimadzu 8400S FTIR spectrophotometer (Kyushu, Japan) and a ESCA 2000 from VG Microtech (London, UK). TGA measurements were tested with the TA Q50 (NewCastle, DE, USA) under a N_2_ atmosphere to confirm the shell thickness of BT@ZnPc. Differential scanning calorimetry (DSC) testing were measured with the TA Instruments DSC Q100 (NewCastle, DE, USA) from 25 to 350 °C at 10 °C/min. The heating rate was 10 °C/min from room temperature to 350 °C. Rheological properties of the composite film are measured by a TA AR-G2 rotational rheometer (NewCastle, DE, USA). TH 2819A precision LCR meter (Changzhou, China) was applied in determining dielectric properties of PEN dielectric composites.

## 3. Results and Discussion

In this paper, PEN and BT nanocomposites with enhanced dielectric properties were obtained by using a ZnPc buffer as the plasticizer. The ZnPc, which was wrapped on the surface of BT, showed excellent compatibility with the PEN matrix which contributed to the enhanced properties of the system. Polyarylene ether nitrile (PEN) whose structure is shown in Scheme 1 was firstly synthesized through nucleophilic aromatic substitution polymerization according to the literature mentioned technique [21,22]. BT@ZnPc was then prepared through the esterification between the carboxyl from the ZnPc-COOH and the hydroxyl on the peripheral of hydrogen peroxide treated BT (BT-OH). Finally, BT@ZnPc was incorporated into the PEN matrix and then the compatibility and the enhanced dielectric properties of the obtained system were investigated.

**Figure 1:**
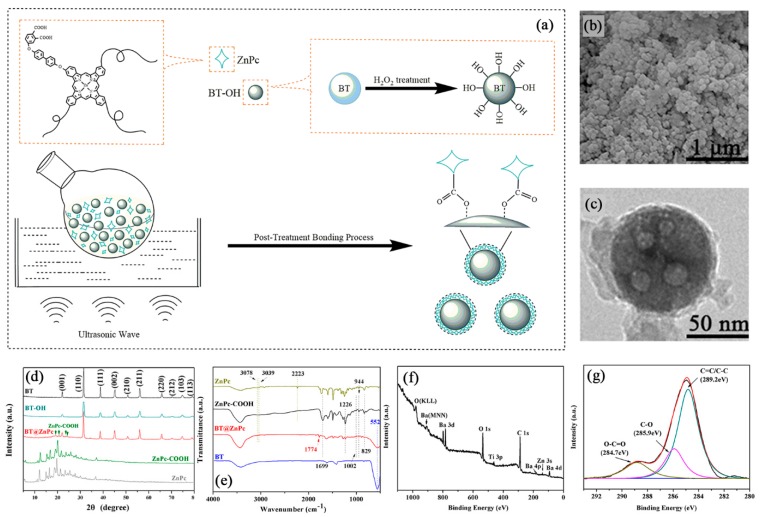
(**a**) Synthetic route for Barium titanate(BT)@ZnPc; (**b**) SEM image of BT@ZnPc; (**c**) TEM image of BT@ZnPc; (**d**) XRD patterns of BT, BT-OH, ZnPc, ZnPc-COOH and BT@ZnPc; (**e**) FTIR spectra of ZnPc, ZnPc-COOH, BT@ZnPc and BT; (**f**) XPS spectrum of BT@ZnPc; and (**g**) XPS C 1s spectrum of BT@ZnPc.

**Figure 2:**
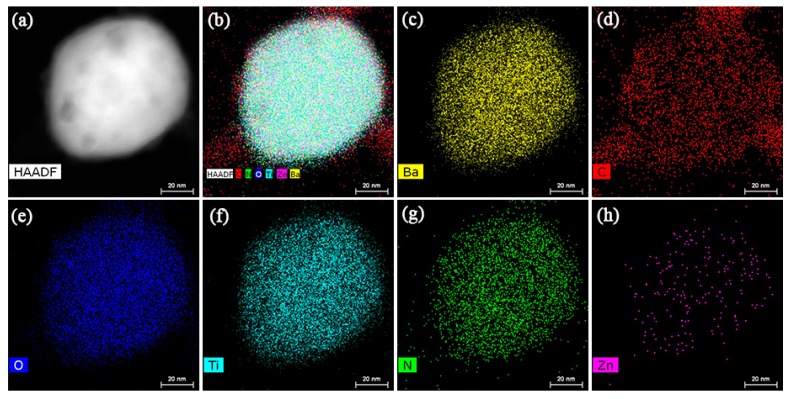
(**a**) STEM images of BT@ZnPc. EDS mapping images of (**b**) all elements, (**c**) Ba, (**d**) C, (**e**) O, (**f**) Ti, (**g**) N, (**h**) Zn.

BT@ZnPc was fabricated according to the scheme shown in Figure 1a. BT-OH was prepared through treating BT with hydrogen peroxide for 6 h, and ZnPc-COOH was synthesized by hydrolyzing ZnPc in NaOH solution and acidulating with HCl afterwards. Then, the BT@ZnPc was prepared through the esterification between the carboxyl from the ZnPc-COOH and the hydroxyl on the peripheral of BT-OH. Morphologies of this fabricated BT@ZnPc were confirmed through TEM and SEM observation. Figure 1b depicts the micro-graph from the BT@ZnPc nanoparticles. It can be seen that the BT-OH coated with ZnPc-COOH exhibited a relatively uniform spherical shape with an average size of 50 nm. Figure 1c presents the TEM micrograph of BT@ZnPc, from which the surface of BT-OH coated with ZnPc-COOH can be clearly observed. The core-shell structured BT@ZnPc was also confirmed by the STEM micrograph and the TEM energy dispersive spectrometer mapping. As shown in Figure 2b, Ba, C, O, Ti, N, and Zn can be observed from the micro-sphere in the figure, indicating the existence of BT-OH and ZnPc-COOH. In addition, the distributions of C, N, and Zn, which come from ZnPc-COOH, are wider than those of Ba and Ti that attributed from BT-OH, demonstrating that the ZnPc-COOH is wrapped on the peripheral of BT-OH and the formation of BT@ZnPc.

The prepared core-shell structured BT@ZnPc was further characterized by XRD, FTIR, and XPS. As shown in Figure 1d, BT shows diffraction patterns at 2θ= 22.1°, 31.4°, 38.8°, 45.2°, 50.8°, 56.1°, 65.7°, 70.3°, 74.7°, and 79.5° which corresponding to the (001), (110), (111), (002), (210), (211), (001), (220), (212), (103), and (113), crystal planes of BT and the BT exhibits cubic phase [26,27]. The BT-OH shows the same diffraction patterns as that of BT (Figure 1d). ZnPc-COOH exhibits a halo peak from 10° to 40°, which is also the same as that of ZnPc [28]. In comparison, BT@ZnPc combines the diffraction patterns of BT-OH and ZnPc-COOH, indicating the existence of the corresponding components in BT@ZnPc and the modification did not change the crystals structure of BT or BT-OH. Figure 1e presents the FTIR spectra of BT-OH, ZnPc, ZnPc-COOH, and BT@ZnPc. As shown in the figure, the absorption peak at 553 cm^−1^ from spectrum of BT-OH results from Ti-O vibration. For ZnPc-COOH, the absorptions occurring at 3078, 3039, 1598, 1003, 944, and 829 cm^−1^ belongs to the absorption peaks of phthalocyanine. The absorption peak appearing at 1227 cm^−1^ indicates the presence of the ether bond. While the characteristic peak located at 1699 and 3450 cm^−1^ can be attributed to the carboxyl group. Besides, the characteristic absorption peak of -CN(2223 cm^−1^) is clearly observed in the curve of ZnPc, but not observed in the curve ofZnPc-COOH, which indicates that the disappearance of characteristic peak of –CN on the FTIR spectrum of ZnPc-COOH, suggesting that all of the –CN has been hydrolyzed.The FTIR spectrum of BT@ZnPc exhibits all the characteristic peaks from the BT-OH and ZnPc-COOH. Besides, the new peak appearing at 1745 cm^−1^ on the spectrum indicates the formation of the ester bond in BT@ZnPc, indicating the reaction between the carboxyl from the ZnPc-COOH and the hydroxyl on the peripheral of BT-OH. XPS was also utilized to characterize the BT@ZnPc. As shown in Figure 1f, C, O, Ti, Zn, and Ba are observed from survey spectrum of BT@ZnPc, indicating the reaction betweenZnPc-COOH and BT-OH. The C 1s spectrum of BT@ZnPc could be quantitatively differentiated into three different carbon species located at 284.7, 285.9, and 289.2 eV, respectively (Figure 1g). These three peaks represent the chemical bonds of the carbon-carbon bond, ether bond, and carbonyl bond, respectively. According to the above characterizations, it can be confirmed that the BT@ZnPc core-shell structured particle has been successfully prepared.

**Figure 3:**
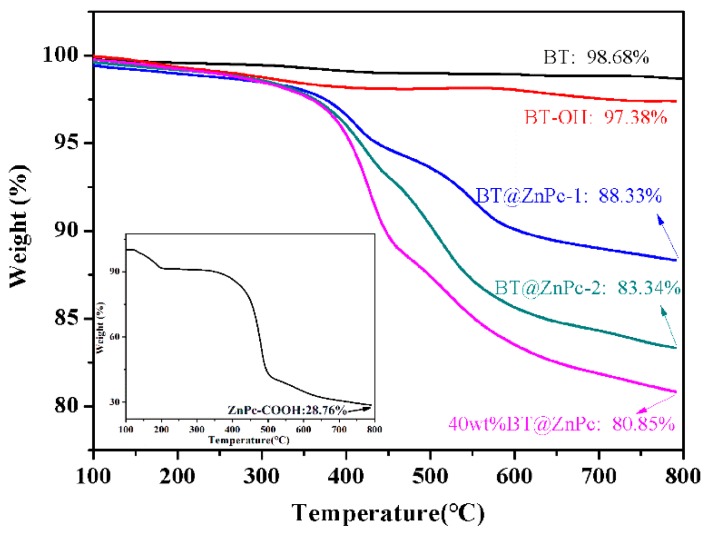
Thermogravimetric analysis (TGA) curves of BT, BT-OH, ZnPc-COOH, BT@ZnPc-1, BT@ZnPc-2 and BT@ZnPc-3. Inset, TGA curve of ZnPc-COOH.

TGA can also be used to characterize the grafting of ZnPc-COOH on the surface of BT-OH. In parallel, the content of ZnPc-COOH can be obtained from the TGA results. When heated to 800 °C in anitrogen atmosphere, BT-OH shows a weight loss of 2.6 wt% (residual weight of 97.4 wt%), as depicted in Figure 3. In comparison, most part of ZnPc-COOH was decomposed at 800 °C and the residual weight was only 28.8 wt% [23]. With the increasing of ZnPc-COOH, the residual weight of BT@ZnPc-1, BT@ZnPc-2, and BT@ZnPc-3 decreases to 88.3, 83.3, and 80.8 wt%, respectively. As a result, the calculated content of ZnPc-COOH in BT@ZnPc-1, BT@ZnPc-2, and BT@ZnPc-3 is 13.3, 20.6, and 24.2 wt%, respectively.

**Figure 4:**
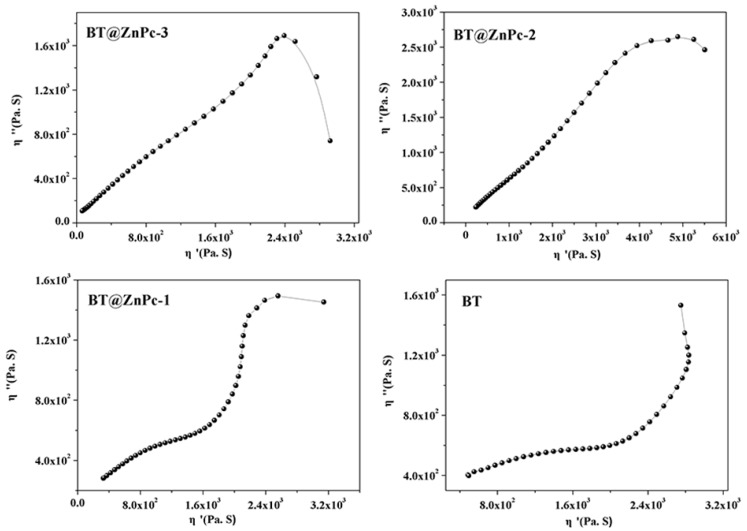
Cole-Cole plot curves of PEN composites with 30 wt% of BT@ZnPc-3; BT@ZnPc-2; BT@ZnPc-1 and BT.

The prepared BT@ZnPc was introduced into the PEN matrix through the solution casting method. Obvious aggregation of BT was observed in PEN due to the poor compatibility between BT and PEN [29]. In this study, the ZnPc was incorporated as the buffer to improve the compatibility between them. The compatibility between BT@ZnPc and PEN was investigated through rotating rheology shears measurement. It is well known that the effect of fillers on the relaxation behavior of polymers can be obtained by studying the viscoelastic behavior of polymer multi-component composites [30,31,32]. Figure 4a–d are Cole-Cole model curves for PEN composites with 30 wt% of BT@ZnPc-1, BT@ZnPc-2, BT@ZnPc-3, and BT, respectively. The Cole-Cole curve of BT@ZnPc-3/PEN is close to the semicircle (Figure 4a). With the ZnPc-COOH content decrease, the curve deviates significantly from the semicircular state (Figure 4b). When BT@ZnPc-1 or BT was used as the filler, the Cole-Cole curve gradually rises in the high viscosity region (low frequency region). This behavior deviating from the semicircular shape trajectory indicates different compatibilities between the filler and the PEN matrix. Since the change in rheological behavior in the low frequency region is due to the contribution of long relaxation time, the increasing at the tail of the BT@ZnPc-1/PEN or BT/PEN Cole-Cole curve indicates that there is a longer relaxation time in the system, which is caused by the poor compatibility between the filler and the substrate in this system. Therefore, the Cole-Cole curves of the PEN composites confirms that the ZnPc-COOH shell in the core-shell structured BT@ZnPc improves the molecular chain motion of PEN, and the content of ZnPc-COOH needs to be higher than 20 wt% to obtain a compatible system.

**Figure 5:**
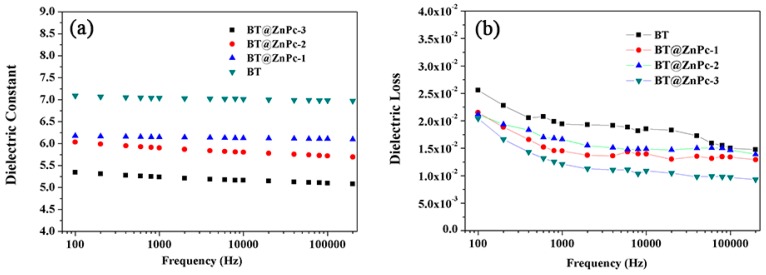
Dielectric constant (**a**) and dielectric loss (**b**) of PEN composites with 30 wt% of BT, BT@ZnPc-1, BT@ZnPc-2, and BT@ZnPc-3.

As a high dielectric constant materials, BT was always utilized to improve the dielectric constant of polymer materials [33]. In this work, the BT was also incorporated into PEN to obtained composites with high dielectric. Figure 5 exhibits the dielectric constant of PEN composite with 30 wt% of BT, BT@ZnPc-1, BT@ZnPc-2 and BT@ZnPc-3. The dielectric constant of PEN is 3.43 at 1 kHz. With the existence of the fillers, the dielectric constant increases obviously. The dielectric constant is 7.19 at 1 kHz for BT/PEN, and it decreases gradually from BT@ZnPc-1/PEN to BT@ZnPc-3/PEN due to the lower dielectric constant of ZnPc-COOH. It is also interesting to see that the dielectric constant of BT@ZnPc-2/PEN is only a little lower than that of BT/PEN. According to the compatibility and dielectric properties of the PEN based composites, the compatibility between the fillers and PEN increases but the dielectric constant decreases, with the thickness of the shell organic layer of the core-shell structure BT@ZnPc increases. Therefore, BT@ZnPc-2, which has a moderate thickness of shell, was selected as the additive for studying the influence of the filler content on the properties of the PEN based composite.

**Figure 6:**
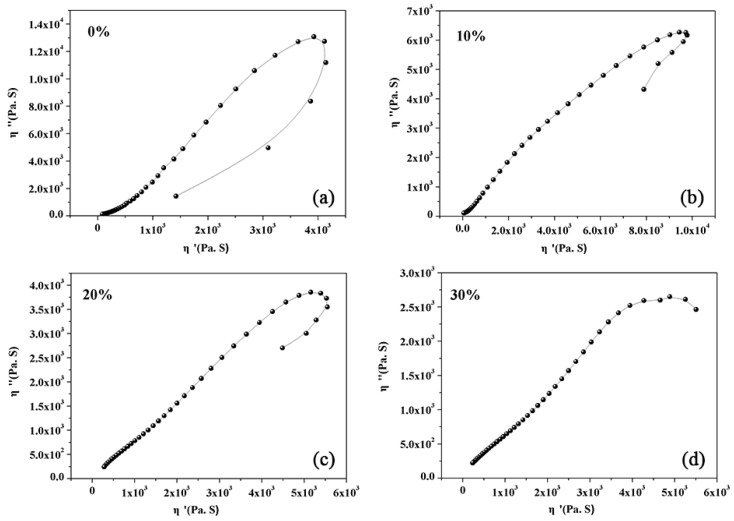
Cole-Cole plot curves of BT@ZnPc-2/PEN composite with 0 wt% (**a**); 10 wt% (**b**); 20 wt% (c) and 30 wt% (**d**) of BT@ZnPc-2.

**Figure 7:**
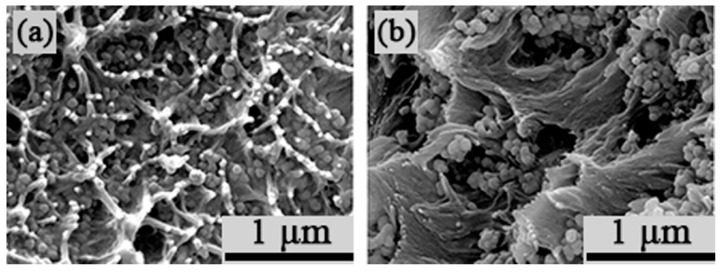
(**a**) SEM image of BT@ZnPc-2/PEN film; (**b**) SEM image of BT@ZnPc-2/PEN film after isothermal annealing at 280 °C for 5 h.

To study the content of BT@ZnPc-2 on the properties of the BT@ZnPc-2/PEN composite, 0, 5, 10, 20, and 30 wt% of BT@ZnPc-2 was introduced into PEN matrix, respectively. The compatibility between BT@ZnPc-2 and PEN was also investigated by dynamic rheological testing, which is often used to describe the distribution of viscoelastic behavior of heterogeneous polymer composites with relaxation time [32]. Figure 6a–d are Cole-Cole plots of BT@ZnPc-2/PEN composite with a BT@ZnPc-2 filler content of 0, 10, 20, and 30 wt% at 330 °C, respectively. It can be clearly found that Cole-Cole curve of pure PEN is a semi-circular arc, indicating that the molecular chain of PEN polymer exhibits almost a complete relaxation state. When the filler content increases, the semicircular arc of the composite gradually decreases. These results indicate that the movement of the PEN polymer chain is limited, suggesting that the long-range relaxation of the PEN molecular chain becomes the main form of relaxation as the filler content increases, which proves that the increase of the filler limits the movement of the PEN molecular chain. On the other hand, the Cole-Cole curves of all the composites maintained a semi-circular arc profile, implying that the excellent compatibility between BT@ZnPc-2 and PEN. To further determine the compatibility between BT@ZnPc-2 and PEN, the scanning electron microscope observation was employed. Figure 7a shows a cross-sectional SEM photograph of a BT@ZnPc-2/PEN dielectric composite with a filler content of 30 wt%. It can be seen from the figure that there is no obvious phase interface between the polymer matrix and the nanoparticles in the composite, indicating that the BT@ZnPc-2 nanoparticles exhibit excellent compatibility with the PEN matrix.

**Figure 8:**
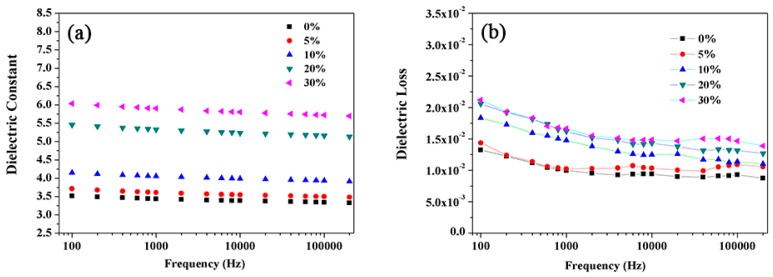
Dielectric constant (**a**) and dielectric loss of BT@ZnPc-2/PEN films with the changing of frequency.

The dielectric properties of the BT@ZnPc-2/PEN composite were further studied in detail. As shown in Figure 8a, the dielectric constant of pure PEN and BT@ZnPc-2/PEN decreases with increasing frequency. The decrement is 5.6%, 5.9%, 5.6%, 6.2%, and 5.3% for PEN composites with 0, 5, 10, 20, and 30 wt% of BT@ZnPc-2, respectively, showing a stable dielectric constant with the changing frequency. In addition, consistent with expectations, the dielectric constant of BT@ZnPc-2/PEN composite increases with the increasing of BT@ZnPc-2 content, which contributes to the high dielectric constant of BT. The dielectric constant of BT@ZnPc-2/PEN with 30 wt% of BT@ZnPc-2 is up to 6.05, with an improvement of 70%. Another important parameter that dielectric materials need to focus on is the dielectric loss of the material. In general, if the dielectric material has a large dielectric loss, part of the stored energy will be consumed in the form of heat or the like during use, which is pernicious for its application. Figure 8b shows the dielectric loss of BT@ZnPc-2/PEN composites. Similar to that of dielectric constant, the dielectric loss of the BT@ZnPc-2/PEN composite decreases with the increasing of frequency and increases with the increasing of BT@ZnPc-2 content. More importantly, the dielectric loss of the BT@ZnPc-2/PEN composite is lower than 0.02 at a frequency higher than 100 Hz, which is resulted from the ZnPc-COOH buffer.

**Figure 9:**
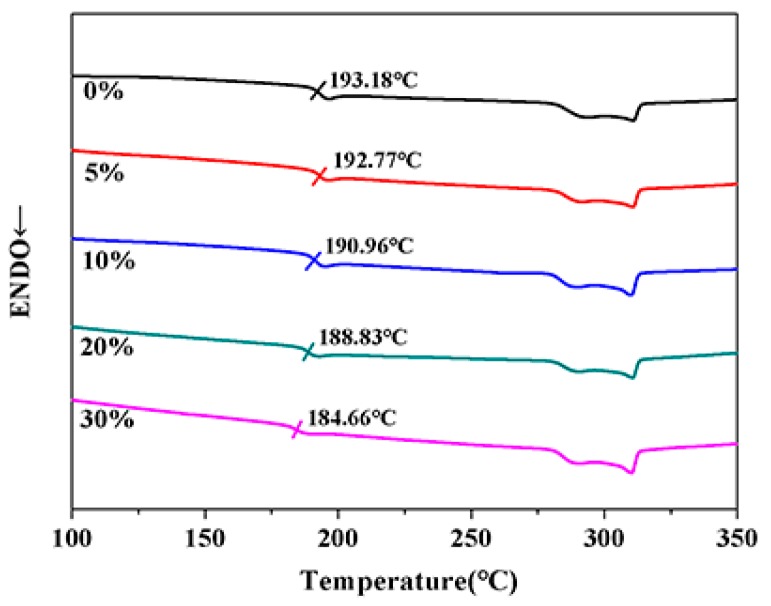
DSC curves of BT@ZnPc-2/PEN films with 0, 5, 10, 20, and 30 wt% of BT@ZnPc-2.

It has been reported that the crystalline of the polymer can improve the dielectric constant and decrease the dielectric loss of the system [34]. As the PEN used in this work contains rigid biphenyl segment (Scheme 1), it can crystallize at temperatures higher than its glass transition temperature. Figure 9 depicts the DSC curves of the BT@ZnPc-2/PEN composite heated from room temperature to 350 °C. As shown in Figure 9, all the BT@ZnPc-2/PEN composites exhibit a melting point during the scan. The double melting points of PEN have been reported by L. Tu et al. who contributed them to the perfect crystals and imperfect crystals [35,36]. As a result, the dielectric properties of the BT@ZnPc-2/PEN composites can be potentially enhanced through isothermal crystallization. In addition, the glass transition temperature of PEN is around 193.2 °C. With the introducing of BT@ZnPc-2, the glass transition temperature of the obtained BT@ZnPc-2/PEN composite decreases a little, which would be caused by the internal plasticization of ZnPc-COOH.

**Figure 10:**
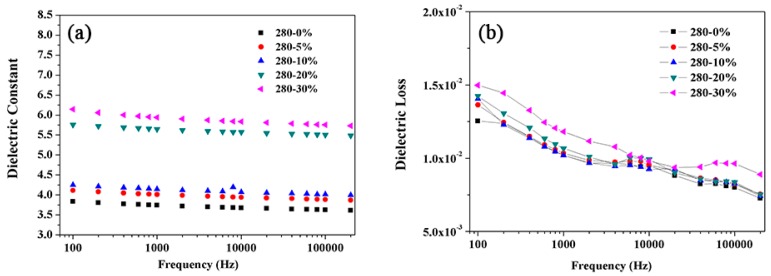
Dielectric constant (**a**) and dielectric loss (**b**) of BT@ZnPc-2/PEN films after isothermal annealing at 280 °C for 5 h.

Figure 10 shows the dielectric constant and dielectric loss of BT@ZnPc-2/PEN composites isothermal annealed at 280 °C for 5 h. After the isothermal annealing, the PEN substrate in the BT@ZnPc-2/PEN composites crystalized, which can be confirmed by the SEM micro-image shown in Figure 7b. As a result, the dielectric constant of each composite has increased compared with the ones that have not been annealed. Specially, the dielectric constant of annealed BT@ZnPc-2/PEN composite with 30 wt% of BT@ZnPc-2 is 5.94 at 1 kHz (Figure 10a). This phenomenon is mainly due to the fact that the crystal matrix formed during the crystallization of PEN will squeeze the filler into the amorphous region, causing the increasing content of filler in the amorphous region, which increases the dielectric constant to a certain portion. Figure 10b shows the dielectric loss of the BT@ZnPc-2/PEN composites annealed at 280 °C. Compared with the original one, the dielectric loss of the composites after crystallization is reduced. The decrease in dielectric loss after crystallization is due to the orderly arrangement of the polymer segments of the PEN matrix, which results in the limited thermal motion of the polymer composite. In addition, the ZnPc-COOH buffer works as an insulating layer covering the barium titanate particles, resulting in excellent compatibility between the PEN matrix and BT particles, which reduces the dielectric loss caused by the interfacial polarization. What is more, the good dispensability of BT@ZnPc particles in the PEN matrix also inhibits the increase of dielectric loss to some extent.

## 4. Conclusions

In summary, polymeric composite dielectric materials were fabricated with BT@ZnPc as the fillers and PEN as the matrix. BT@ZnPc were prepared through the esterification between BT-OH and ZnPc-COOH and then characterized by SEM, TEM, EDS mapping, XRD, FTIR, and XPS. TGA result indicates that the content of ZnPc-COOH is 13.3, 20.6, and 24.2 wt% for BT@ZnPc-1, BT@ZnPc-2, and BT@ZnPc-3, respectively. BT@ZnPc/PEN composites with high dielectric constant and low dielectric loss were prepared by a solution casting method. The test of the rotary shear rheometer proved that the BT@ZnPc particles are compatible with the PEN matrix due to the existence of ZnPc-COOH buffer. The wt % BT@ZnPc-2 of 0, 5, 10, 20, and 30 wt% of was introduced into the PEN matrix to study the content of BT@ZnPc-2 on the properties of the BT@ZnPc-2/PEN composite. Results showed that the BT@ZnPc/PEN composite film exhibits high dielectric constant and low dielectric loss. In addition, dielectric constant of the BT@ZnPc/PEN composites can be further improved while the dielectric loss of them was depressed after isothermal annealation at 280 °C for 5 h. The results indicate BT@ZnPc/PEN composite has potential application prospects in the field of dielectric materials.
